# Geological and engineering appraisal of hydraulic frac sand in some Egyptian localities as a proppant of oil well drilling

**DOI:** 10.1016/j.heliyon.2022.e10233

**Published:** 2022-08-15

**Authors:** Gaber M.A. Wahab, Gamal El-Din A. Ibrahim, Amna A.M. Abdel Wahab

**Affiliations:** Egyptian Petroleum Research Institute, Exploration Department, Nasr City, Cairo, Egypt

**Keywords:** Frac sand, Hydraulic fracturing, Silica sand, Unconventional reservoirs, Horizontal drilling Western desert, Eastern desert, Egypt

## Abstract

Sand that comprises high purity silica grains, in large percent, is of the best naturally occurring grains that can be used as proppants during hydraulic fracturing processes. Proppants are used to increase formations' permeability; to increase reservoirs' productivity, or to reopen plays and utilize unconventional reservoirs. The potentiality of these grains to be used as frac proppants is determined according to certain physical, mechanical, petrographical and chemical evaluations that include particle size analysis, acid solubility, turbidity, bulk density, crush resistance and hardness, sphericity and roundness, mineral and chemical composition. This study shows the evaluation of the silica sand samples collected from Malha Formation in Wadi El Dakhal, Eastern Desert; Naqus Formation in Wadi Qena, Eastern Desert; and Bahariya Formation at Gabal El-Dist area in Bahariya Oasis, Western Desert, Egypt. The samples were examined according to frac sand international standards. The results showed the potentiality of the tested samples to be utilized as frac sand proppants. Wadi El-Dakhal and Wadi Qena studied areas possess very promising samples for frac sand production. But, the quality of Wadi El-Dakhal samples is somewhat better than that of Wadi Qena samples. The samples of Gabal El-Dist in Bahariya Oasis are relatively less to achieve the requirements; however, they can be utilized in shallow depths.

The assessment testing of the studied samples revealed a good results and verifying the standard requirements, where SiO_2_ content is 89.1% in Wadi Qena, 99.3 % in Wadi Dakhal and 78.1% in Gebel El Dist, crush resistance at 5000 psi gives fine percent 4.71 W.Q, 6.78 W.D, and 14.11 B.O, turbidity readings raining from 100.5 to 133.25 NTU, the grain roundness are rounded to sub rounded, and grain size distribution range is 30/50 to 40/70 grading (710 um to 210 um).

## 1 Introduction

In 2020, the global frac sand market amounted to 7.27 Billion US dollars that shows the current increase in the demand for frac sand. This increase is mainly due to the increase in the exploration activity and dependence on unconventional hydrocarbon resources.

Now, unconventional hydrocarbon resources are the future of hydrocarbon production as they represent the main global non-renewable energy reserves, the unconventional gas reserves are represent eight times the conventional reserves, so the demand for unconventional resources increased that increased the demand for unconventional exploration and production techniques; increasing the demand for horizontal drilling that in turn increased the demand for hydraulic fracturing and the amount of frac sand needed during the drilling operations. Modern hydraulic fracturing is used to produce hydrocarbon from unconventional reservoirs with low permeability including shale, tight sandstones, and coal beds also low to moderate permeability sandstone, limestone and dolostone reservoirs. The horizontal drilling depends mainly on hydraulic fracturing processes where these reservoir layers are of very low permeability reaching <0.1 mD that they will never produce at an economic production rate without hydraulic fracturing as they are not commercial even by acidizing and formation destroying. Hydraulic fracturing has also been used in the completion and increasing the recovery of different types of wells and formations with respect to different ranges of criteria. It is also now being used to reopen plays that have been capped to enhance recovery. Given the increase in hydraulic fracturing processes, the demand for frac sand will continue to increase where about 1000 ton of frac sand is needed for each hydraulic fracturing process.

Egypt is possessing high quantities of pure silica sand reserves and also light brownish sand dunes reserves that can be used as industrial frac sand. According to the degree of compatibility of their physical, mechanical, petrographical, and chemical properties with the frac sand international standards, some of these reserves can be used in industrial frac sand production. Despite the availability of these kinds of sand deposits in Egypt; they aren't well utilized, studied and evaluated in the frac sand industry. Also, according to the United States Geological Survey (USGS) Egypt's mineral year book statistics, the production of industrial sand in Egypt is either decreasing or nearly constant from 2011 to 2015. It is more expensive in case of importing sand proppants as the transportation cost increases its overall cost; therefore locating a source near to the areas of petroleum production is greatly beneficial on all aspects **(**America Petroleum Institute, 2014; Imarc, 2021; Liang et al., 2016; Salameh, 2015; Taib, 2019 & Mary et al, 2015).

Many current research's give the priority to the new Ceramic Proppant materials, which is produced of ceramic particle, ceramic proppant used as frac has a high level of fracturing intensity, it's used for oilfield as down hole proppant. Ceramic proppant composition consists of a good quality bauxite and other additive material, granulating and calcining to improve the strength and density of final product. It's a substitute of natural quartz sand, glass sand, metallic sand and other frac materials; Ceramic proppant is more expensive comparing to the other products e.g natural silica sand. This research find an appropriate low price and good quality alternative proppant types, which can be utilized with good efficiency in increasing the productivity of oil and natural gas (V. P. de Campos1 et al., 2018).

## 2 Materials and methods

### 2.1 Field sampling

**Figure 1:**
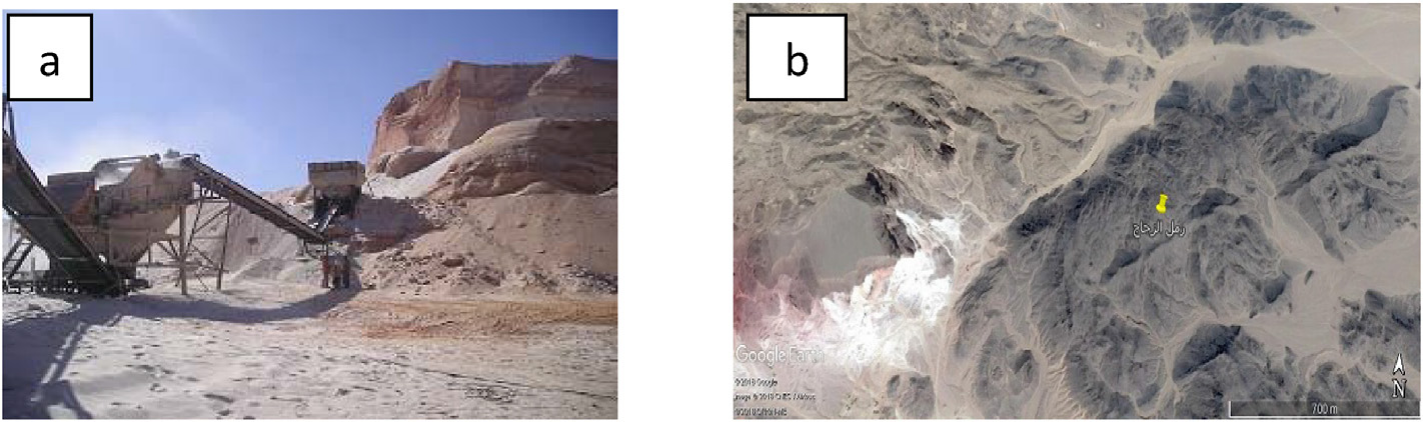
Photographs of Wadi Dakhal silica sand (a) Al Motehta quarry (b) Pit-rock quarry.

Geological field trips were conducted for collecting thirty samples from the three studied localities. The studied areas includes Wadi El-Dakhal in Zaafarana area, Eastern Desert, Egypt that is outlined by latitudes 28°40′54.3″ N and 28°45′ N and longitudes 32°26′35.2″ E and 32°30′ E, Wadi Qena in Eastern Desert, outlined by latitudes 27°41′53.9″ N and 27°59′ N and longitudes 32°30′ E and 33° E and Bahariya Oasis is outlined by latitudes 28°00′ N and 29° N and longitudes 28°48′ E and 29°09′ E in Western Desert, Egypt (Figure 1). The samples were collected and classified for laboratory testing and characterization. The tested samples represent Malha Formation in Wadi El-Dakhal, Naqus Formation in Wadi Qena and Bahariya Formation at Gabal El-Dist area.

The sand deposits of Wadi El Dakhal are considered as raw material for the white sand industrial purposes and. the Malha Formation is the Cretaceous rock units bearing the white sands (Boulos et al., 2017; Hesham et al., 2017 and Metwally et al., 2016).

### 2.2 Particle size distribution

The dry sieving method with screens placed at one-phi interval was the particle size distribution method conducted for the collected sand samples. Calibrated U.S sieves of sieve mesh sizes falling in the ranges of the typical proppant or gravel-pack size designations as defined in **(**ASTM- E11- 2017, Table 1). The size designations used were 6/12, 8/16, 12/18, 12/20, 16/20, 16/30, 20/40, 30/50, 40/60, 40/70, 70/140 size (API RP 19C 2008 & ISO 13503 -2- 2006 & Mark, 2007).

**Table 1:** The standard sieve sizes as defined in (ASTM- E11- 2017).

Sieve-opening sizes μm											
	3350/1700	2360/1180	1700/1000	1700/850	1180/850	1180/600	850/425	600/300	425/250	425/212	212/106
Typical frac sand proppants' size designations											
	6/12	8/16	12/18	12/20	16/20	16/30	20/40	30/50	40/60	40/70	70/140
Stack of ASTM sieves											
$1^{\text{st}}$ primary sieve **(bold type)**	4	6	8	8	12	12	16	20	30	30	50
	**6**	**8**	**12**	**12**	**16**	**16**	**20**	**30**	**40**	**40**	**70**
	8	10	14	14	18	18	25	35	45	45	80
$2^{\text{nd}}$ primary sieve **(bold type)**	10	12	16	16	**20**	20	30	40	50	50	100
	**12**	14	**18**	18	25	25	35	45	**60**	60	120
	14	**16**	20	**20**	30	**30**	**40**	**50**	70	**70**	**140**
	16	20	30	30	40	40	50	70	100	100	200
	Pan	Pan	Pan	Pan	Pan	Pan	Pan	Pan	Pan	Pan	Pan

### 2.3 Acid solubility (HCl/HF)

The preferred method of testing acid solubility is using a solution of 12:3 hydrochloric acid (HCI): hydrochloric fluoric acid (HF) (i.e. 12 % by mass of HCI and 3 % by mass of HF) at room temperature. The solubility of a proppant in 12:3 HCI: HF is an indication of the amount of soluble materials (i.e. carbonates, Feldspars, iron oxides, clays, etc) present in the proppant. This test indicates the samples degree of solubility to work in acidic environments as well (API 19C 2008 & ISO 13503-2- 2006**)**.

### 2.4 Turbidity (absence of clay & silt)

To describe silt and clay size particulate content, turbidity was measured using UV-Visible Spectrophotometer model JASCO V-570 that is calibrated with wavelength adjusted at 450 nm, using the conversion chart calibration curve of turbidity in NTU (or FTU) verses absorbance. The turbidity of the samples should be 250 FTU and/or NTU or less (API 19C 2008 & ISO 13503-2- 2006).

### 2.5 Bulk density

There are two important physical properties for frac sand raw materials includes: bulk density and apparent density. Bulk density describes the mass of proppant that fills a unit volume and includes both proppant and porosity. It is used to determine the mass of a proppant required to fill a fracture of layers containing oil/gas. Apparent density is measured with a low-viscosity fluid that wets the particle surface and includes the pore space inaccessible to the fluid. On the other hand, the absolute density excludes pores that can be in the proppant as well as void spaces between proppant particles (American National Standards Institute B74.4 1992; Gaber et al, 2021).

### 2.6 Crush resistance

The test measure the amount of proppant crushed at a given stress. The test was done under stress of 5000 psi and the maximum percentage of fines produced from the samples was determined, by using the equation: ${\text{m}}_{\text{pan}}$ = $\frac{{\text{m}}_{\text{pan}}}{{\text{m}}_{\text{s}}}$ × 100.

Where: ***m***_pan_ is the mass of fines generated material in the pan, expressed in grams; ***m***_s_ is the mass of proppant used as the sample aliquot, expressed in grams.

### 2.7 Hardness

Hardness was measured with Lm500 hardness tester at Egyptian Petroleum Research Institute, to determine their degree of resistance to deformation, scratching, abrasion and cutting. (Reade Advanced Materials, 2020). Hardness is a property by which minerals may be described relative to a Mohs scale standard of 10 minerals. The degree of hardness is determined by observing the comparative ease or difficulty with which one mineral is scratched by another or by a steel tool.

### 2.8 Sphericity and roundness

Visual determination of sphericity and roundness is generally the most used method. The tested grains were placed on a suitable background, spread to a one-particle-thickness layer. About twenty grains were randomly selected and examined in the field view through 40 times magnification light binocular microscope comparing them to (Krumbein and Sloss, 1963). They have to be determined for every individual grain to get an average representative value of the whole sand sample (API 58, 1995, API 19C 2008; Gaber and Ibrahim, 2021).

### 2.9 X-ray fluorescence

X-ray fluorescence provides qualitative and quantitative analysis. It was used to identify the samples' constituents including SiO_2_, TiO_2_, Al_2_O_3_, Fe_2_O_3_, MgO, CaO, Na_2_O, K_2_O, P_2_O_5_, Cl, and LOI. The X–Ray fluorescence test was carried out for the tested samples using Rigaku's Supermini200 wavelength dispersive X-ray fluorescence spectrometer.

### 2.10 X-ray diffraction

X-ray diffraction was used as a semi-quantitative method to determine the mineral components of the frac sand samples. The XRD test was conducted using X'Pert³ Powder which is PANalytical's newest X-ray diffraction system based on the fully renewed X'Pert platform.

## 3 Results

### 3.1 Sieve analysis

**Figure 2:**
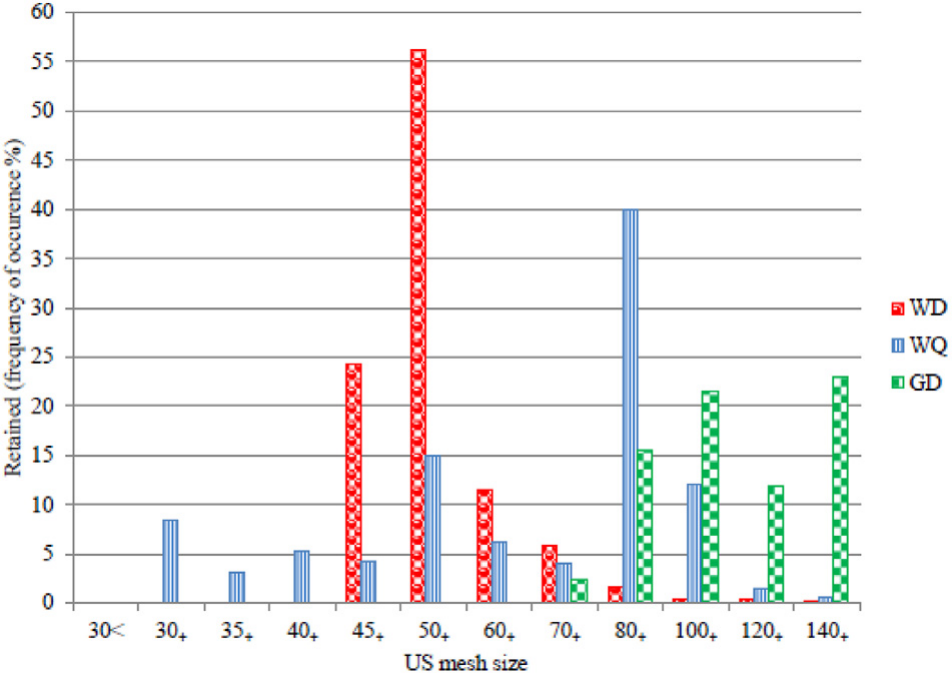
Histogram showing comparison of sieve analysis for three localities WD, WQ & BO.

According to the sieve analysis results of the test samples, the Wadi El-Dakhal are typically of 40/70 proppant size designation, where more than 90 percent passed the 40 US mesh and was retained on the 70 US mesh, and not more than 0.1 percent of the total sample is larger than the 30 US mesh and not more than 1 percent of the total sample is smaller than the 100 US mesh as indicated in Table 2. The Wadi Qena samples not represent a single proppant size designation specifically; none of them has at least 90 percent of its mass plotted in between any of the $1^{\text{st}}$ primary and $2^{\text{nd}}$ primary designated sieves and 70/140 is the nearest size designation they can reach as illustrated in Table 3. The "”100 mesh” refer to 50/140 or 40/140 frac sand products as per (Frac Sand Specifications, 2019 & API 19 C, 2008). The Bahariya Oasis not represent a single proppant size designation. They mainly passed the 70 US mesh and were retained on the 140 US mesh, with pan fraction ranging from 24.2 to 27.7% as indicated in Table 4. The mean diameter (${\text{d}}_{\text{av}}$) was calculated in millimeters to be used for classification the proppant utilized in hydraulic fracturing beside the mesh-size, where ${\text{d}}_{\text{av}}$ = Σn.d/Σn and n·d is the product of mid-size diameter (d) multiplied by frequency of occurrence as illustrated in (Figure 2).

**Table 2:** Mean diameter calculation parameters and the sieve analysis results showing the average distribution of grain sizes of the Wadi El-Dakhal (WD) samples.

US mesh size	Retained %: frequency of occurrence (n) % by mass	Cumulative %	Passing %	US mesh size interval	Particle-size interval mm	Mid-size diameter (d) mm	n·d
40 mesh	0	0	100	35 to 40	0.5 to 0.425	0.4625	0
45 mesh	24.2	24.2	75.8	40 to 45	0.425 to 0.355	0.39	9.438
50 mesh	56.1	80.3	19.7	45 to 50	0.355 to 0.3	0.3275	18.3727
60 mesh	11.5	91.8	8.2	50 to 60	0.3 to 0.25	0.275	3.1625
70 mesh	5.8	97.6	2.4	60 to 70	0.25 to 0.212	0.231	1.3398
80 mesh	1.6	99.2	0.8	70 to 80	0.212 to 0.18	0.196	0.3136
100 mesh	0.4	99.6	0.4	80 to 100	0.18 to 0.149	0.1645	0.0658
120 mesh	0.3	99.9	0.1	100 to 120	0.149 to 0.125	0.137	0.0411
140 mesh	0.1	100	0	120 to 140	0.125 to 0.106	0.1155	0.01155

**Table 3:** Mean diameter calculation parameters and the sieve analysis results showing the average distribution of grain sizes of Wadi Qena (WQ) samples.

US mesh size	Retained %: frequency of occurrence (n) % by mass	Cumulative %	Passing %	US mesh size interval	Particle-size interval mm	Mid-size diameter (d) mm	n·d
25 mesh	0	0	100	20 to 25	0.85 to 0.71	0.78	0
30 mesh	8.4	8.4	91.6	25 to 30	0.71 to 0.6	0.655	5.502
35 mesh	3.1	11.5	88.5	30 to 35	0.6 to 0.5	0.55	1.705
40 mesh	5.2	16.7	83.3	35 to 40	0.5 to 0.425	0.4625	2.405
45 mesh	4.1	20.8	79.2	40 to 45	0.425 to 0.355	0.39	1.599
50 mesh	15	35.8	64.2	45 to 50	0.355 to 0.3	0.3275	4.9125
60 mesh	6.2	42	58	50 to 60	0.3 to 0.25	0.275	1.705
70 mesh	4	46	54	60 to 70	0.25 to 0.212	0.231	0.924
80 mesh	40	86	14	70 to 80	0.212 to 0.18	0.196	7.84
100 mesh	12	98	2	80 to 100	0.18 to 0.149	0.1645	1.974
120 mesh	1.5	99.5	0.5	100 to 120	0.149 to 0.125	0.137	0.2055
140 mesh	0.5	100	0	120 to 140	0.125 to 0.106	0.1155	0.05775

**Table 4:** Mean diameter calculation parameters and the sieve analysis results showing the average distribution of grain sizes of Bahariya Oasis (BO) samples.

US mesh size	Retained %: frequency of occurrence (n) % by mass	Cumulative %	Passing %	US mesh size interval	Particle-size interval mm	Mid-size diameter (d) mm	n·d
40 mesh	0	0	100	35 to 40	0.5 to 0.425	0.4625	0
45 mesh	0	0	100	40 to 45	0.425 to 0.355	0.39	0
50 mesh	0	0	100	45 to 50	0.355 to 0.3	0.3275	0
60 mesh	0	0	100	50 to 60	0.3 to 0.25	0.275	0
70 mesh	2.4	2.4	97.6	60 to 70	0.25 to 0.212	0.231	0.5544
80 mesh	15.5	17.9	82.1	70 to 80	0.212 to 0.18	0.196	3.038
100 mesh	21.6	39.5	60.5	80 to 100	0.18 to 0.149	0.1645	3.5532
120 mesh	11.8	51.3	48.7	100 to 120	0.149 to 0.125	0.137	1.6166
140 mesh	23	74.3	25.7	120 to 140	0.125 to 0.106	0.1155	2.6565

For Wadi El-Dakhal samples, the average mean diameter (${\text{d}}_{\text{av}}$) = Σn.d/Σn = 32.7451/100 = 0.327 mm Table 2. Wadi Qena samples, the average mean diameter (${\text{d}}_{\text{av}}$) = Σn.d/Σn = 28.83/100 = 0.2883 mm Table 3. Bahariya Oasis samples, the average mean diameter (${\text{d}}_{\text{av}}$) = Σn.d/Σn = 11.4187/74.3 = 0.1536 mm as indicated in Table 4.

### 3.2 Acid solubility

The acid solubility results of Wadi El-Dakhal tested samples, Wadi Qena tested samples and Bahariya Oasis tested samples are listed in Table 5. The recommended standard acid solubility values are affected by the samples' particle sizes, and their silica composition. According to (API 19C 2008 & ISO 13503-2- 2006), the size ranging from 6/12 to 30/50 proppant sizes should have maximum solubility of 2% or less by weight. While the proppants of 40/70 to 70/140 mesh sizes should have maximum solubility of 3% or less by weight.

**Table 5:** Acid solubility percent of the tested samples.

Area	Sample code	Acid solubility percent	Average
Wadi El-Dakhal	WD1	1.17	2.28
	WD2	3.42	
	WD3	1.33	
	WD4	3.22	
Wadi Qena	WQ1	11.05	10.00
	WQ2	9	
	WQ3	10.1	
	WQ4	9.86	
Bahariya Oasis	BO1	4.3	12.31
	BO2	11.8	
	BO3	16.9	
	BO4	16.25	

Wadi El-Dakhal samples average acid solubility is 2.28 %, samples fall within the 3% limit of 40/70 size proppants. Wadi Qena samples don't represent a specific frac sand proppant size designation. 70/140 is the nearest size designation it can reach after beneficiation it can also be regarded as a "100″ mesh; frac particularly 40/140 size. The average acid solubility of its samples is 10%; so all samples don't fall within the 3% limit. Bahariya Oasis tested samples don't represent a frac proppant size designation. They mainly passed the 70 US mesh and were retained on either the 120 US mesh or 140 US mesh, with pan fraction ranging from 24.2 to 27.7%. The average acid solubility of its samples is 12.31%, exceeding the maximum 3% limit.

### 3.3 Turbidity

The turbidity test showed that the water phase was clear enough to distinguish the identification label on the bottle for all tested samples of Wadi El-Dakhal, Wadi Qena and Baharyia Oasis samples; the samples were considered clean and suitable for use as recorded in Table 6. The turbidity values of Wadi El-Dakhal samples ranges from 111 to 117 NTU. The turbidity values of Wadi Qena samples range from 96 to 103 NTU. The turbidity values of Bahariya Oasis samples range from 127 to 139 NTU. The turbidity of the samples should be 250 FTU and/or NTU or less. The maximum turbidity values for Wadi El-Dakhal samples, Wadi Qena samples and Bahariya Oasis samples are 117 NTU, 103 NTU and 139 NTU respectively. All the tested samples' turbidity values comply with the standards and none of them exceeded the 250 NTU limit.

**Table 6:** Turbidity of tested samples in nephelometric turbidity units.

Area	Sample code	Turbidity (NTU)	Average
Wadi El-Dakhal	WD1	117	114.75
	WD2	111	
	WD3	117	
	WD4	114	
Wadi Qena	WQ1	96	100.5
	WQ2	103	
	WQ3	103	
	WQ4	100	
Bahariya Oasis	BO1	139	133.25
	BO2	127	
	BO3	132	
	BO4	135	

### 3.4 Bulk and apparent density

The bulk and apparent density results of studied samples showed that the average bulk density for Wadi El-Dakhal sample is 1.66 g/cm³, while the apparent density is 2 g/cm³, Wadi Qena sample bulk density is 1.69 g/cm³, while the apparent density is 2.12 g/cm³, the bulk density of Bahariya Oasis sample is 1.71 g/cm³, while the apparent density is 2.3 g/cm³.

### 3.5 Crush resistance

The crush resistance results recorded in Table 7 indicates the amount of proppant material crushed from the silica sand samples at specified load of 5000 psi. The percentage of material crushed does not exceed 8% for the 40/70 size designation and does not exceed 6% for the 70/140 size designation (API 19C, 2008, API 60, 1995 & ISO 13503-2- 2006).

**Table 7:** Crush resistance values of the tested samples under 5000 psi stress.

Area	Sample code	Fines percent	Average
Wadi El-Dakhal	WD1	8	6.78
	WD2	7.1	
	WD3	6.8	
	WD4	5.22	
Wadi Qena	WQ1	4.1	4.708
	WQ2	4.23	
	WQ3	6	
	WQ4	4.5	
Bahariya Oasis	BO1	11.32	14.105
	BO2	11.7	
	BO3	15.1	
	BO4	18.3	

The Wadi El-Dakhal samples of 40/70 size designation, While Wadi Qena samples of 70/140 size designation or "100 mesh” frac sand particularly 40/140 size. Therefore only the material retained on the 140 US mesh sieve was used. Bahariya Oasis samples were evaluated in the crush resistance test as a product of 70/140 size designation as well. Wadi El-Dakhal samples result is less than 8% and Wadi Qena samples less than 6%, while Bahariya Oasis samples result is about 14% and considered weaker and might need resin coating to improve their strength.

### 3.6 Hardness

The results of hardness test of collected sample are indicated in Table 8. The average hardness value of Wadi El-Dakhal sample is 6.615, Wadi Qena samples is 6.897, and Gabal El-Dist sample is 6.21. The results indicate that the all types are hard enough and can resist the impingement in tight layers.

**Table 8:** Hardness of Bahariya Oasis (BO) samples.

Area	Sample code	Hardness	Average
Wadi El-Dakhal	WD1	6.71	6.615
	WD2	6.62	
	WD3	6.5	
	WD4	6.63	
Wadi Qena	WQ1	6.92	6.897
	WQ2	6.94	
	WQ3	6.82	
	WQ4	6.91	
Bahariya Oasis	BO1	6.53	6.21
	BO2	6.22	
	BO3	6.05	
	BO4	6.04	

### 3.7 Sphericity and roundness

**Figure 3:**
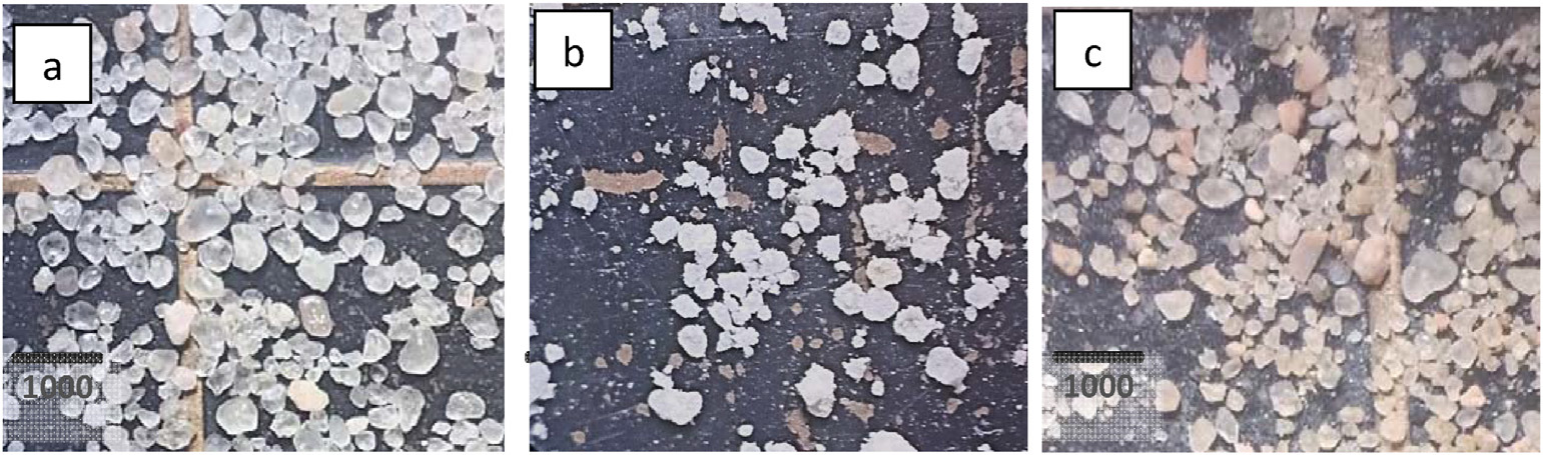
Photomicrographs under light binocular microscope (a) WD1 (b) WQ4 (c) BO2 (40× magnification).

**Figure 4:**
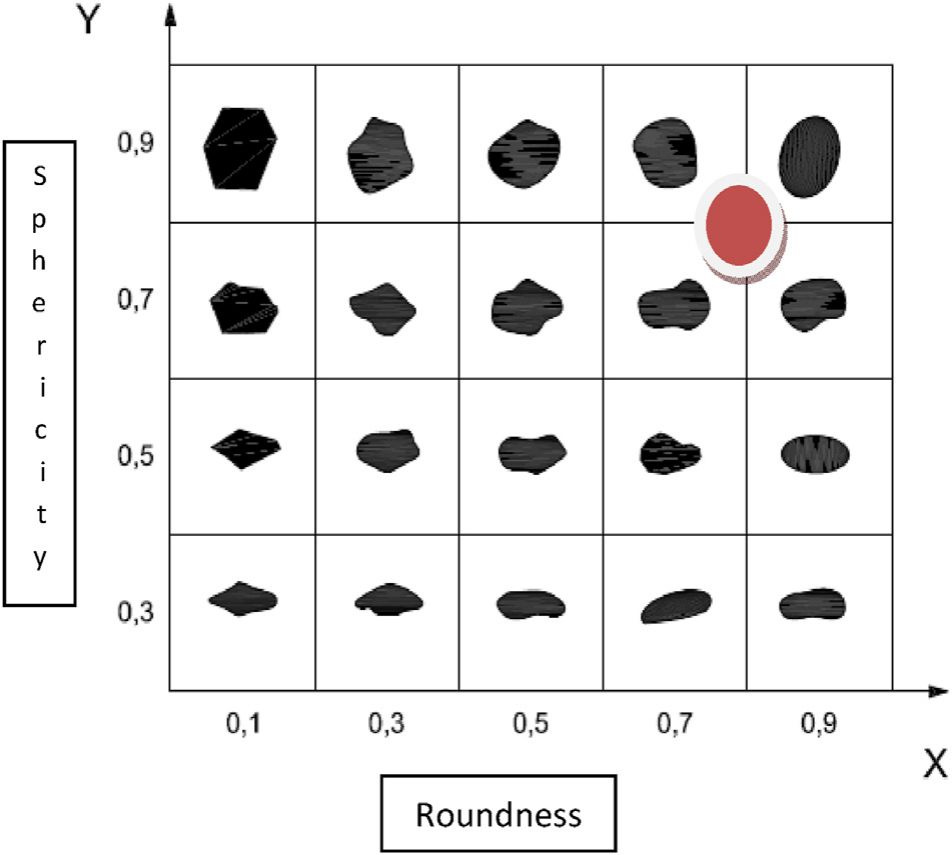
Plotting of studied sample sphericity and roundness diagram of frac sand.

The samples were evaluated by examining under 40 times magnification microscope and comparing with Krumbein and Sloss chart (Figures 3 and 4). According to the recommended hydraulic fracturing (API 19C, 2008; ISO 13503-2, 2006), shall be 0.6 or higher. The results recorded in Table 9.

**Table 9:** Sphericity and roundness of tested sample.

Area	Sample code	Sphericity	Roundness	Average sphericity	Average roundness
Wadi El-Dakhal	WD1	0.7	0.6	0.675	0.65
	WD2	0.7	0.7		
	WD3	0.6	0.7		
	WD4	0.7	0.6		
Wadi Qena	WQ1	0.6	0.5	0.65	0.55
	WQ2	0.6	0.5		
	WQ3	0.7	0.5		
	WQ4	0.7	0.7		
Bahariya Oasis	BO1	0.7	0.7	0.675	0.675
	BO2	0.7	0.6		
	BO3	0.7	0.7		
	BO4	0.6	0.7		

### 3.8 X–ray fluorescence

The chemical analysis of collected samples indicates the minimum SiO2 is 68.2 % at Baharyia and maximum SiO2 is 99.3 % at wadi El Dakhal as illustrated in Table 10.

**Table 10:** Chemical analysis results for the tested samples.

Area	Sample code	Elements wt.%										
		SiO_2_	TiO_2_	Al_2_O_3_	Fe_2_O_3_	MgO	CaO	Na_2_O	K_2_O	P_2_O_5_	Cl	LOI
Wadi El-Dakhl	WD1	99.3	-	-	-	-	0.042	-	-	0.0098	0.037	0.45
	WD2	99	-	-	0.049	-	0.024	-	-	0.009	0.026	0.7
	WD3	98.2	-	0.388	0.080	-	0.084	-	0.01	0.015	0.053	0.818
	WD4	85.1	0.096	6.13	0.813	0.480	1.62	1.79	1.41	0.081	0.033	2.21
Wadi Qena	WQ1	87	-	10.1	0.071	-	0.213	-	-	0.046	0.037	2.3
	WQ2	89.1	-	9.09	0.088	-	0.096	-	0.023	0.062	0.027	1.34
	WQ3	87.8	0.280	9.31	0.088	-	0.427	-	0.007	0.068	0.03	1.47
	WQ4	88.4	0.323	9.09	0.134	-	0.204	-	0.034	0.106	0.044	1.44
Bahariya Oasis	BO1	95.4	-	1.77	0.326	0.167	0.686	0.403	0.497	0.049	0.016	0.5
	BO2	78.1	0.522	5.07	3.23	0.76	6.24	0.84	1.56	0.151	0.181	2.4
	BO3	69.1	0.952	6.99	4.91	1.33	8.65	0.86	1.67	0.207	0.232	3.54
	BO4	68.2	0.933	5.28	4.35	1.03	10	1.03	1.68	0.18	1.14	3.98

### 3.9 X–ray diffraction

XRD test was conducted using X'Pert³ Powder which is PANalytical's newest X-ray diffraction system.

**Figure 5:**
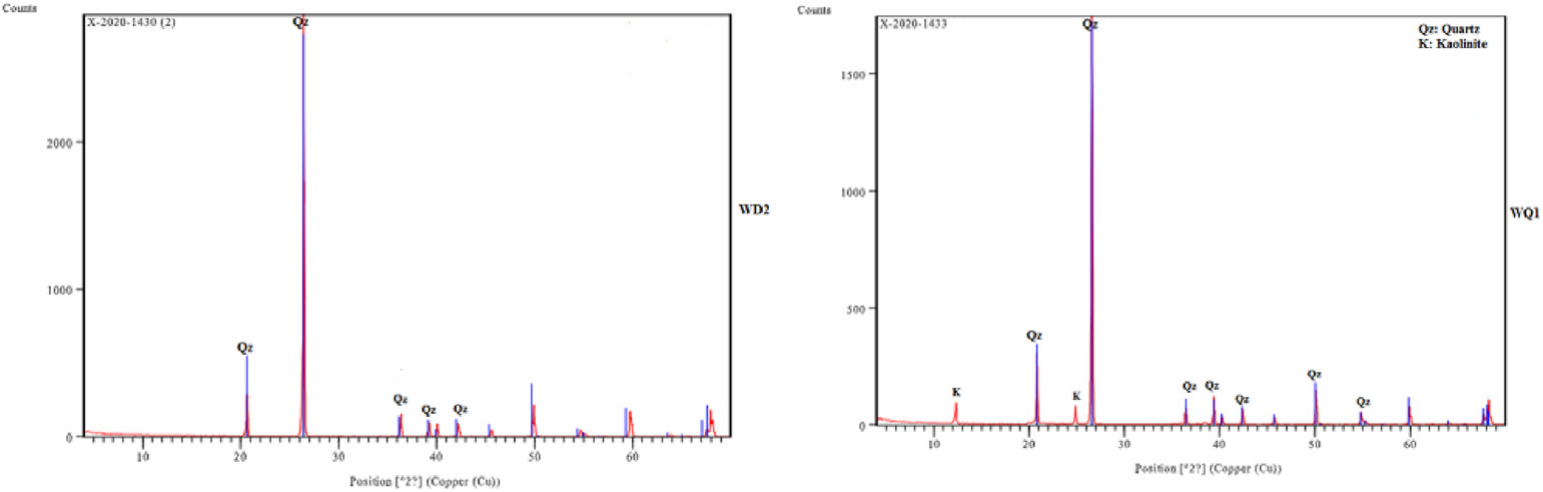
X-ray Diffraction pattern (a) WD2 (b) WQ1.

XRD measured in rang from 4.01 to 69.99 2 Ɵ with a step size of 0.02° (Cu radiation). The diffraction patterns indicate that the predominant is quartz in Wadi El-Dakhal, Wadi Qena and Gabel El Dist samples. There are some kaolinite, calcite and feldspare minerals as shown in (Figure 5).

### 3.10 Good friability (not solid)

Friable deposits of silica sand, poorly cemented sandstone are most desirable in frac sand manufacture (Runkel et al., 2012), loose silica sand not required to be blasted during excavation or crushing during mining processing. Friable silica frac sand easily mined by large excavators (Maslowski, 2012). The crushing process of consolidated silica sand may cause fracturing of grain size and increase the angularity and reduce the roundness of sand grain size.

### 3.11 Opencast mining to reduce cost

The Wadi Dakhal and Wadi Qena samples are exposed on the ground level with <1 m overburden slightly consolidated, having the advantage of easy mining and low cost operations, meantime both studied areas are located near by the infrastructure facilities.

## 4 Discussion

The physical, chemical and mechanical properties of three different localities obtained from Eastern and Western desert in Egypt was presented with respect to frac sand as a lowest price and moderately to high efficiency. Sieve Analysis, Bulk Density, Apparent density, Sphericity and Roundness, Acid Solubility, Turbidity and Crush Resistance, XRF and XRD analysis; were evaluated as per international standard of oil fields. The three localities have a bulk density of 1.66–1.71 g/cm³; Hardness 6.21 to 6.61; Sphericity and Roundness of 0.55–0.675; Acid Solubility was found in the range of 2.28–12.31 %; while the Turbidity ranging between 100.5 to 114.75 FTU; samples crush resistance friction at 5000psi was varying from 4.708 to 14.105 % and SiO_2_ content ranging from 68.2 to 99.30. Comparison of the obtained results with the reference value presented by API RP 19C indicates that the two localities (Wadi el Dakhale and Wadi Qena) samples are compatible with the requirements for sand proppant. The result of Baharyia sample not verifies the standard requirements.

## 5 Conclusion

This research evaluated the collected samples from Malha Formation, Wadi El-Dakhal, Eastern Desert, Naqus Formation, Wadi Qena, Eastern Desert and Bahariya Formation at Gabal El-Dist, Bahariya Oasis, Western Desert; The tested samples were examined according to the API, ASTM, and ISO requirements. They were analyzed to measure grain size and shape, acid solubility, turbidity, bulk density, hardness, resistance to crushing, and silica content. The results concluded that Wadi El-Dakhal and Wadi Qena areas possess very promising for production of frac silica sand. The samples selected from Gabal El-Dist, Bahariya Oasis are less in quality and need some beneficiation.Therefore, Bahariya Oasis tested samples can be utilized in hydraulic fracturing applications in shallow depths. This study indicates some positive conclusions:

- Egypt possesses billons of white and brown sand quantities mined by open cast method of low cost.
- Wadi El Dakhal sand consists of high purity SiO_2_ with 99.3%; while the Wadi Qena sand has 89.10 %SiO_2_.
- Crush resistance results recorded a very good value at 5000 psi.
- Eastern Desert sand are distinguished with appropriate; grain roundness, grain size grading and crush resistance.
- The grain size analysis of studied samples in Wadi Dakhal and Wadi Qena indicates that more than 90% of frac sand sizes are type 20/40, 30/50 and 40/70, the obtained results comply with the standard requirements.
- The study recommends to use both types of sand as frac sand, due to proper physical, chemical and mechanical properties, both studied areas are located nearby the Gulf of Suez oil fields.

## Declarations

### Author contribution

Gaber, M. A. Wahab: Conceived and designed the experiments; Analyzed and interpreted the data; Wrote the paper.

Gamal El-Din A. Ibrahim: Analyzed and interpreted the data.

Amna A. M. Abdel Wahab: Performed the experiments; Contributed reagents, materials, analysis tools or data.

### Funding

This research did not receive any specific grant from funding agencies in the public, commercial, or not-for-profit sectors.

### Data availability

The data that has been used is confidential.

### Declaration of interest

The authors declare no conflict of interest.

### Additional information

No additional information is available for this paper.

## References

[bib1] America Petroleum Institute (2014). http://www.api.org/oil-and-natural-gas-overview/exploration-and-production/hydraulic-fracturing/hydraulic-fracturing-qa.aspx.

[bib2] American National Standards Institute B74.4 (1992). Procedure for Bulk Density of Abrasive Grains.

[bib3] America Petroleum Institute Recommended Practices 58 (1995). Recommended Practices for Testing Sand Used in Gravel-Packing Operations.

[bib4] America petroleum Institute recommended practices 60 (1995). Recommended Practices for Testing High Strength Proppants Used in Hydraulic Fracturing Operations.

[bib5] America Petroleum Institute Recommended Practices 19C (2008).

[bib6] American Society for Testing and Materials E11 (2017).

[bib7] Boulos T.R., Ahmed Y., Mohamed B.M., Ibrahim S.S. (2017). High quality fused silica from Egyptian silica sand concentrate. Int. J. Sci. Eng. Invest..

[bib8] de Campos1 V.P.P., Sansone E.C., e Silva G.F.B.L. (2018).

[bib9] (2020). Frac Sand Spec General Information, 2019: Lonquist-Frac Sand Services.

[bib10] Gaber M. Wahab, Ibrahim G. (2021). Characterization of some Egyptian white sand and dunes for utilization as hydraulic fracturing sand for tight oil well layers. Geolog. Surv. Egypt.

[bib11] Hesham A.H., Mohamed M.A., Ali I.A. (2017). Evaluation of white silica sand in North Eastern Desert, Egypt. Int. J. Sci. Eng. Res..

[bib12] Imarc (2021). https://www.imarcgroup.com/frac-sand-market.

[bib13] ISO 13503-2, 2006: Petroleum and Natural Gas Industries Completion Fluids and Materials: Measurement of Properties of Proppants Used in Hydraulic Fracturing and Gravel-Packing Operations.Geneva, Switzerland. International Standard Organization.

[bib14] Krumbein W., Sloss L. (1963).

[bib15] Liang F., Sayed M., Al-Muntasheri A., Chang F., Li L. (2016). A comprehensive review on proppant technologies. Petroleum.

[bib16] Mark D. (2007).

[bib17] Mary E.B., Anna B.W. (2015).

[bib18] Maslowski A. (2012). Well Servicing Magazine.

[bib19] Metwally S.A., Abouziena H.F., Bedour M.H. (2016). Biological method in the stabilization of sand dunes using the ornamental plants and woody trees: a review article. JIPBS.

[bib21] Reade advanced materials (2020). https://www.reade.com/reade-resources/reference.

[bib22] Runkel A.C., Stenberg J.R. (2012).

[bib23] Salameh M. (2015). Reference Module in Earth Systems and Environmental Sciences.

[bib24] Taib M. (2019).

